# Derived Relations and Attentional Bias for Near-Misses in Slot Machines

**DOI:** 10.1007/s10899-025-10420-z

**Published:** 2025-08-01

**Authors:** Leigh D. Grant, Steve Provost

**Affiliations:** 1 School of Psychology, Charles Sturt University, Port Macquarie, Australia; 2 Southern Cross University, Coffs Harbour, Australia

**Keywords:** Attentional bias, Gambling, Equivalence classes, Near win, Near miss, Slot machines

## Abstract

This study investigates the influence of derived relations on attentional bias toward near-misses in slot machine gambling, expanding on the consistent findings of the effect in gambling research. We aimed to replicate earlier findings by examining how learning to associate near-misses with a "loss" affects attentional bias to gambling-related stimuli. The study employed an experimental design in which 24 recreational gamblers were randomly assigned to one of two conditions in a relational training task: one group was trained to associate near-misses with the concept of "loss," the other with "almost." Participants engaged in a simulated slot machine game while their eye movements were tracked from which attentional bias for near-miss slot-machine outcomes was derived from eye-tracking data. The results revealed that participants who learned to associate near-misses with "loss" exhibited a significant reduction in their attentional bias for near-miss outcomes compared to those who learned to associate near-misses as being an "almost" gambling result. These findings further support problem gambling research indicating that near-misses are a potent event capable of capturing and maintaining attention, aligning with cognitive bias theories in gambling. Moreover, the study provides additional support for incentive-sensitization theory and suggests potential applications for targeted interventions in gambling disorders.

Problem gambling is an ongoing public health concern. It affects between 0.5% and 2% of the adult population, yet these individuals contribute disproportionately—often 40% or more of total revenue (Armstrong & Carroll, 2017). Why do some individuals persist in gambling when it strains their finances, fractures their relationships, and undermines their wellbeing? Answers lie partly in the sophisticated design of modern gambling technologies, which exploit vulnerabilities in human cognition and decision-making (Rickwood et al., 2010). Electronic gaming machines (EGMs) represent a particularly insidious form of gambling technology. These devices, with their easy access, rapid play, and careful engineering, provide gamblers with an absorbing form of play that can exploit common cognitive biases (Griffiths, 1993, 1999; Schüll, 2012). Central to EGMs problem gambling potential is the near-miss effect: outcomes that seem tantalizingly close to a win yet are, in fact, losses.

In gambling, the near-miss effect occurs when a player experiences a loss that feels close to a win (Reid, 1986). Unlike other losses, near-misses can either represent a frustrating near-victory or suggest that a win is within reach, encouraging further betting (Dixon et al., 2013). EGMs, with their multiple reels, weighted reels, and potential for programmed near-miss outcomes, are particularly susceptible to this phenomenon (Chóliz, 2010). Derived relations, the ability to make novel connections based on previously learned information, play a significant role in the development and maintenance of problem gambling (Dixon et al., 2009). Derived verbal relations can drive preference for slot machines in general and reduce the perceived difference between near-misses and wins (Dixon et al., 2009; Tan et al., 2015). Attentional bias, the preferential focus on gambling-related stimuli, can intensify gambling urges, contribute to cognitive distortions, and perpetuate problem gambling behavior (Ciccarelli et al., 2016). The degree of attentional bias for gambling stimuli compared to neutral stimuli can be seen as an index of the sensitization of the dopaminergic reward system for near-miss event reinforcement, offering a more objective measure than the self-report rating scales typically used in near-miss studies (Grant & Bowling, 2015). The purpose of the current study was to integrate findings from these earlier lines of research by demonstrating how derived relations influence the effect of near-misses on gambling behavior, as measured by attentional bias.

## The Near-Miss Effect and Electronic Games Machines

In everyday life, a near-miss is a good sign. Picture yourself crumpling paper into a ball, aiming for the bin. It hits the rim and bounces out. Next time, you'll throw harder. You learn and adapt. However, betting on EGMs is different. Each spin is like a coin flip—the past doesn't matter (Reid, 1986). Research demonstrates that gamblers often misperceive near-misses as indicators of skill improvement or impending success, despite slot machine outcomes being entirely chance-based (Dixon et al., 2013; Griffiths, 1999). This cognitive distortion reflects a misapplication of real-world learning principles to random gambling contexts (Clark et al., 2009).

The near-miss effect in gambling refers to the cognitive distortion that occurs when an outcome that closely resembles a win, despite being a loss, is perceived by a player that they are almost succeeding. This near-win experience, while objectively a loss, heightens arousal and excitement, thereby reinforcing the player's motivation to continue gambling (Griffiths, 1999; MacLaren et al., 2014). This effect operates through the illusion of control, where the player erroneously believes that they are getting closer to winning, and the sustained hope that a win is imminent (Côté et al., 2003). The near-miss effect can significantly influence gambling behavior by activating the brain's reward pathways in a manner similar to actual wins, releasing dopamine and generating feelings of pleasure and excitement (Robinson & Berridge, 2008). This heightened arousal, coupled with the false sense of skill or control, encourages players to continue gambling (Côté et al., 2003), often leading to extended duration of betting and increased overall money wagered (read: gambling losses).

EGMs are intricately designed to exploit near-miss outcomes, significantly increasing a player's susceptibility to this cognitive bias. The multiple reels and paylines typical of EGMs are engineered to create numerous opportunities for near-miss combinations, where symbols align closely to winning patterns but fall short, evoking a sense of "almost winning." This sensation is further amplified by weighted reels, where certain symbols are programmed to appear more frequently than others, heightening the likelihood of near-miss outcomes. For example, less valuable symbols may often appear alongside winning symbols, creating the illusion of being close to a win (Harrigan & Dixon, 2010). Furthermore, modern EGMs have introduced the concept of partial wins—where players receive a return that is less than their original stake—designed to masquerade losses as wins (Harrigan et al., 2011). For instance, a return of 10 cents on a $1.00 bet is often accompanied by celebratory sounds and visual feedback similar to those of a real win, rendering the loss nearly indistinguishable from a victory (Côté et al., 2003; MacLaren et al., 2014). This deceptive practice not only obscures the actual financial loss but also strengthens the psychological impact of near-misses, reinforcing the drive to keep gambling despite mounting losses (Turner et al., 2006). In essence, the design and programming of EGMs are intentionally crafted to exploit the near-miss effect, a key factor in their addictive potential (Chóliz, 2010; Rickwood et al., 2010).

## Derived Relations in Gambling

Derived relations in gambling, as understood within behavioral psychology and learning theory, refer to connections between stimuli that develop without direct training or reinforcement (Dixon et al., 2009, 2013). These relations extend beyond explicitly learned connections, allowing individuals to make novel inferences based on previously acquired information (Griffiths, 1999). In the context of EGMs, derived relations manifest in several notable ways. For instance, stimulus generalisation occurs when a player, having experienced wins or positive emotions on a particular EGM, generalizes these positive associations to other EGMs with similar features or themes, thereby increasing their likelihood of engaging with those machines despite no direct experience with them (Habib & Dixon, 2010). Transitive inference is another influencing factor, where a player who associates a specific EGM with a rewarding experience at a particular casino may infer that other EGMs in the same venue will be similarly rewarding, thus promoting further play (Giroux & Ladouceur, 2006). Additionally, equivalence relations can form when a gambler associates specific symbols or sounds with winning, leading them to perceive other similar stimuli on different EGMs as potentially rewarding, even in the absence of previous wins (Heron, 2013). Furthermore, contextual cues within the gambling environment, such as witnessing other players winning or the ambient atmosphere of a casino, can become associated with positive outcomes, further driving engagement with EGMs. These derived relations underscore the complex, often subconscious processes that influence gambling behavior, setting the stage for understanding how specific patterns, such as the near-miss effect, can further impact player decisions (Chóliz, 2010).

The influence of derived verbal relations on gambling behavior has been a significant area of research in understanding how players make decisions in slot machine gambling. Zlomke and Dixon (2006) conducted a seminal study on verbal relations in slot machine gambling, investigating how players' preferences could be influenced by the association of colors with specific verbal cues. In the initial phase, recreational gamblers played randomly on either a yellow or blue machine. Participants were then trained using a matching-to-sample task to implicitly associate the yellow cue with a “greater than” condition (e.g., $50 > $30) and the blue cue with a “less than” condition (e.g., $20 < $40). When asked to play again, participants showed a marked preference for the yellow machine, suggesting that these derived verbal relations significantly influenced their choices. Building on this foundation, Dixon et al. (2009) explored the impact of verbal relations further, using a similar methodology. Participants were divided into two groups: one associated an abstract symbol with the word “loss” and a near-miss image, while the other linked the symbol with the word “almost” and the same near-miss image. Although the training did not directly connect "loss" and "near-miss," participants who associated “loss” with near-miss rated near-misses as being less like a win, whereas those associating “almost” with near-miss rated them as more like a win. However, the reliance on self-reported ratings in this study raised concerns about potential demand characteristics. To address this limitation, Tan et al. (2015) adopted an experimental approach using objective measures, replicating Dixon et al.’s (2009) procedures. Their findings confirmed that participants who learned “almost = near-miss” were more likely to choose a machine with near-miss outcomes than those who learned “loss = near-miss,” thereby providing robust evidence that derived verbal relations can indeed influence gambling behavior. This study also underscored the value of objective measures in assessing slot machine preferences. Together, these studies highlight the significant role of verbal associations in shaping a gambler's decision to keep playing.

## Attentional Bias in Gambling

Attentional bias refers to an individual’s preference for certain stimuli at the expense of others and plays a crucial role in gambling (Ciccarelli et al., 2016). Studies consistently show that individuals prone to gambling tend to fixate on gambling-related cues, such as flashing lights or the sound of slot machines, while overlooking other aspects of their environment. A comprehensive review by Farr et al. (2023), analyzing 22 studies on the subject, confirmed that attentional bias is prevalent in individuals with gambling problems, irrespective of the assessment method employed. Notably, the intensity of this bias correlated directly with the severity of gambling issues.

Attentional bias manifests as a heightened awareness of gambling-related cues (Ciccarelli et al., 2016). For instance, a gambler might be instantly attracted to the flashing lights of a slot machine or the clinking of coins, while overlooking other, less conspicuous elements of their surroundings. Once engaged, they may find it challenging to disengage, leading to a prolonged fixation on gambling-related stimuli. Further complicating matters, this bias can lead to interpretive biases, where ambiguous stimuli are construed in a way that reinforces gambling-related thoughts and expectations, such as misinterpreting a random sequence of events as a sign of an imminent win (Farr et al., 2023; Hønsi et al., 2013).

This attentional bias is not merely an incidental observation; it is a critical cognitive factor deeply implicated in the development and perpetuation of problem gambling. By amplifying the salience and allure of gambling-related stimuli, it heightens the likelihood of individuals engaging in gambling behavior. Moreover, it can impede their ability to resist gambling urges by constantly drawing their attention towards gambling cues (Farr et al., 2023; Hønsi et al., 2013). It also contributes to the formation and reinforcement of cognitive distortions and irrational beliefs about gambling, such as the illusion of control or the gambler's fallacy, which further exacerbate problematic gambling behavior (Brevers et al., 2014).

Several underlying mechanisms are believed to contribute to attentional bias in gambling. Individuals susceptible to problem gambling may exhibit heightened reward sensitivity, making them more likely to focus on stimuli associated with potential gains (Zlomke & Dixon, 2006). Repeated exposure to gambling-related stimuli and their association with wins or near-misses can establish strong conditioned responses, further increasing their attention-grabbing power (Dixon et al., 2011). Lastly, gamblers may develop cognitive schemas related to gambling, which selectively guide their attention towards relevant information while filtering out irrelevant information. These cognitive distortions can reinforce existing biases and make it even more difficult for individuals to disengage from gambling-related thoughts and behaviors (Toneatto et al., 1998).

Researchers have increasingly turned to eye-tracking as a method to investigate attentional bias in gamblers, providing a nuanced understanding of how individuals engage with gambling-related stimuli (Farr et al., 2023). This technique allows for the continuous and direct measurement of visual attention by recording eye movements, including saccades and fixations, which are grounded in the hypothesis that eye gaze reflects cognitive focus. Eye-movement data is particularly reliable, as it captures the immediate interaction between visual input and cognitive processing, yielding objective and quantitative insights into a person's motivated attention toward salient cues (Hønsi et al., 2013). In typical studies, participants view a series of images that include both gambling-related and neutral stimuli, with increased gaze duration on gambling images serving as an indicator of attentional bias (Brevers et al., 2011; Grant & Bowling, 2015; McGrath et al., 2018). Additionally, the analysis of initial saccades—rapid eye movements occurring within 20 to 200 ms of stimulus onset—provides further evidence of implicit processing, as these movements often occur automatically (Awh, Belopolsky, & Theeuwes, 2012). Thus, eye-tracking emerges as a vital tool for examining the cognitive distortions associated with gambling behavior, paving the way for deeper exploration into the mechanisms underlying attentional biases.

## The Current Study

EGMs pose a significant risk for problem gambling due to their ability to exploit the'near-miss' effect, a cognitive distortion where a loss that closely resembles a win encourages further play. Previous research has demonstrated that derived relations, or learned associations, can influence the perception of near-misses and gambling behavior. However, these studies have primarily relied on self-report measures, which can be susceptible to demand characteristics and subjective biases. The present study aims to address this limitation by replicating the earlier near-miss effect findings using a more objective measure of preferences for near wins: attentional bias.

Gambling-related attentional bias—preferential focus on gambling-related stimuli, has been consistently linked to problem gambling. It can intensify gambling urges, contribute to cognitive distortions, and perpetuate problematic gambling behavior. Furthermore, the degree of attentional bias for gambling stimuli can be seen as an index of the sensitization of the dopaminergic reward system for near-miss event reinforcement. By examining attentional bias, we can gain a more objective understanding of how derived relations influence the perception of near-misses and their subsequent impact on gambling behavior.

This study hypothesizes that individuals who acquire a derived relation between near-wins and “loss” will exhibit a reduced attentional bias for slot machine displays that represent near-misses compared to those who acquire a derived relation between near-wins and “almost.” By testing the effect of derived relations on attentional bias, we can provide more robust evidence for the influence of learned associations on gambling behavior and contribute to the development of more effective prevention and intervention strategies for problem gambling.

## Method

### Participants

Participants included 24 people recruited with convenience sampling from university students and the wider community. Participants were screened using the Consumption Screen for Problem Gambling (CSPG; Rockloff, 2012), a brief four-item screening tool designed to identify individuals at risk for gambling problems. The sample comprised of 14 Males (*M*_*age*_ = 33.1, *SD* = 8.48) and 10 Females (*M*_*age*_ = 39.0, *SD* = 7.79) who volunteered to take part in response to either a flyer posted on licensed club message boards (community), broadcast email (university), or by word of mouth. Participants provided written informed consent. Interested participants with scores on the CSPG categorizing them as non-gamblers were excluded. All participants scored between 1 and 3 on the CSPG; therefore, the sample was classified as recreational gamblers.

### Materials

**The Consumption Screen for Problem Gambling** (CSPG; Rockloff, 2012) was administered to all participants to classify gambling frequency into three categories: non-gambler (excluded from analysis), recreational gambler, or excessive gambler (likely experiencing symptoms of problem gambling). The CSPG is a brief, four-item screening tool designed to identify individuals at risk of gambling-related harm. It was modelled on the ‘Alcohol Use Disorders Identification Test—Consumption’ and serves as its conceptual analogue.

The CSPG comprises three core items assessing the frequency and quantity of gambling behavior over the past 12 months. Respondents answer using a five-point Likert scale: never, less than monthly, monthly, weekly, or daily to almost daily. Example items include: “How often did you gamble in the past 12 months?” and “How often did you spend more than two hours gambling on a single occasion?” Scores range from 0 to 15. A score of 0 denotes a non-gambler; scores between 1 and 3 indicate recreational gambling; scores of 4 or more signify excessive gambling and warrant further screening for problem gambling.

Unlike other gambling assessments, the CSPG focuses solely on consumption (i.e., how often and how long individuals gamble) rather than on gambling-related harm. The CSPG has strong psychometric properties: the probability that a randomly selected case of problem gambling will have a higher score than a randomly selected non-case is 98%. During validation, a cut-off score of 4 or above correctly identified all cases of problem gambling, while only 7.3% of non-problem gamblers scored in this range (sensitivity = 100%; specificity = 92.7%).

### Apparatus

All experimental tasks were completed on Dell computers running Windows 8 operating system with 22″ LCD monitors set at 1680 × 1050 screen resolution. Experimental eye-tracking tasks were programmed in Visual Basic. Verbal training procedure was programmed in e-Prime.

### Stimulus Presentation and Procedure

Participants played the simulated slot machine game twice. First, at the beginning of participation and the second time was immediately after the verbal training task. To enhance ecological validity while maintaining ethical standards, participants were provided with $100 in virtual credits at the beginning of each slot machine session. They were informed that their virtual balance would be tracked throughout the session and that their goal was to have as high a balance as they can. Participants will then be able to see how they ranked compared to other (deidentified) participants in the study. This approach maintains gambling-relevant social motivation without exposing participants to actual financial risk, consistent with ethical guidelines for gambling research (Rockloff & Greer, 2011). Participants could choose between making a small hypothetical “bet” ($0.30) or a large hypothetical “bet” ($1.00) per spin, with wins and losses affecting their virtual balance displayed prominently on screen. The virtual credit and ranking systems have been validated in previous gambling research as effectively eliciting gambling-relevant behaviors and physiological responses comparable to real-money gambling (Ladouceur & Sévigny, 2005; Rockloff & Greer, 2011).

After the bet was placed, the screen was replaced with the word SPINNING in 30-point uppercase ‘Algerian’ font from MS Word. This screen was displayed for 1000 ms and was included to activate participant’s eye-movements in the center of the screen. After 1 s the screen displayed an image of a slot machine outcome, which would be either a win (three identical images across the centre); a near-miss (two identical images across the centre with a third misaligned); or a loss (three different images across the centre line).

The frequency of outcomes per block was set to 18 wins, 36 near-misses, and 78 losses. This ratio equates to 13.6% for wins which is similar to the Australian legal requirement for electronic games machine returns ($0.85–0.90 return for every $1.00 played). During the display of the outcome image, the participant’s eye-movements were recorded. After 2000 ms, the display returned to the bet screen for the beginning of the next trial. Each trial was approximately 4 secs long for a total of 132 trials, which were presented in random order. The task took approximately 10 min to complete.

Between the two simulated slot machine tasks, participants were randomly assigned to one of two conditions in a relational training task: one group was trained to associate near-misses with the concept of "loss," the other with "almost." This derived relations task, which included multiple matching-to-sample trials, replicated the procedure used in Tan et al. (2015). The following has been adapted from their article with variations included when required.

Each trial began with three comparison stimuli presented below another stimulus (the sample). Participants were instructed to click with the computer mouse on one of the three comparison stimuli in response to the sample. Participants were told that different auditory and visual feedback would accompany each response and indicate whether their response was incorrect or correct. Participants were also instructed that they should keep selecting from the comparison stimuli until correct and to “do your best on each trial”. A correct choice was accompanied with multiple high-pitched ascending tones that altogether lasted for 1200 ms: the sound used an open source “slot machine win” noise downloaded from the Internet. At the same time as this auditory feedback, the word CORRECT, written in green, flashed on the screen for 500 ms. Incorrect choices resulted in multiple low-pitched descending tones that altogether lasted for 1005 ms: the incorrect sound also used an open source “slot machine lose” noise downloaded from the Internet. Accompanying the incorrect auditory feedback, written in red, the word WRONG flashed on the screen for 500 ms.

**Fig. 1:**
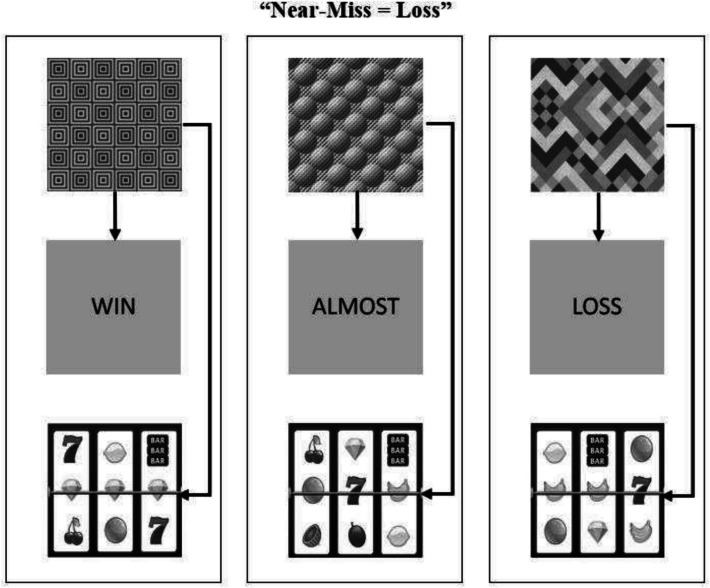
The protocol for training relations in the “near-miss = loss” condition. The arrows in each panel represent the directly trained relations among stimuli

**Fig. 2:**
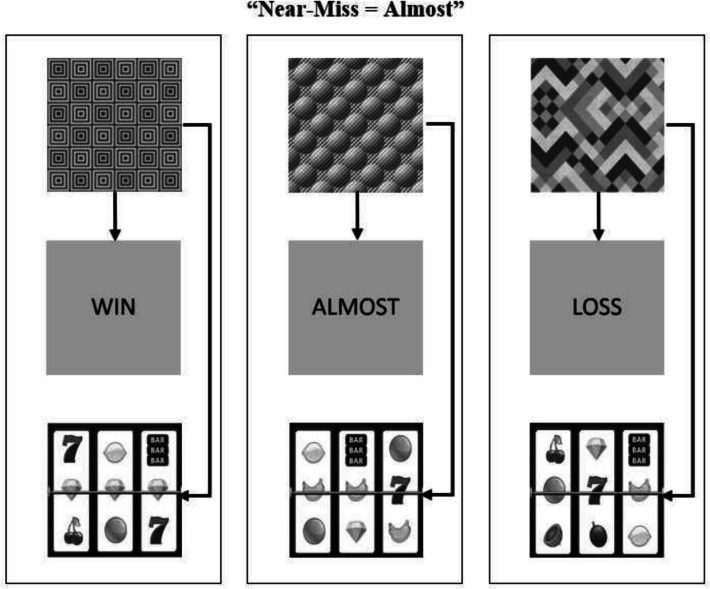
The protocol for training relations in the “near-miss = almost” condition. The arrows in each panel represent the directly trained relations among stimuli

See Figs. 1 and 2 for an example of the three sets of stimuli that were used (A, B, or C). The three comparison stimuli, shown across the bottom of the screen, were drawn from the same set be it A, B, or C. The sample stimulus at the top-centre of the screen was drawn from one of the three sets. For the A stimuli, abstract greyscale images of geometric shapes downloaded from the Internet were used. The B stimuli consisted of grey squares the same size as the A stimulus dimensions with the words “LOSS”, “ALMOST”, and “WIN” printed in black uppercase letters. Finally, the C stimuli used greyscale images of nine different slot machine outcomes that could be grouped in one of three categories (3 × win, 3 × near-miss, or 3 × loss).

Figures 1 and 2 show a diagram of the relations trained for each group. One group of participants (the “near-miss = almost” condition) received training on three stimulus relations: 1) the word “win” was in the same class as the image of a slot machine win (three identical symbols aligning across the middle row); 2) the word almost was in the same class as the image of a slot machine near-miss (two identical symbols on the middle row with one identical symbol offset); 3) the word “loss was in the same class as the image of an outright slot machine loss (nine random symbols). The other group of participants (the “near-miss = loss” condition”) received similar training. However, the almost and loss conditions were reversed: 1) the word “win” was in the same class as the image of a slot machine win (three identical symbols aligning across the middle row); 2) the word almost was in the same class as the image of an outright slot machine loss (nine random symbols; 3) the word “loss was in the same class as the image of a slot machine near-miss (two identical symbols on the middle row with one identical symbol offset).

Three blocks of trials were administered for the training. First, a 16-trial block had the A stimuli as the sample, with the B stimuli as comparisons (A-B training). Second, a 16-trial block again had the A stimuli as the sample, but this time the C stimuli were used for the comparison (A-C training). The third phase was a 36-trial block that presented interspersed trials from the first two blocks (18 trials from the A‒B block, 18 trials from the A‒C block). Participants completed training blocks until they exceeded 88% accuracy criterion for each block (16/18 or 32/36). This criterion is consistent with both Dixon et al. (2009) and Tan et al. (2015) who also required that participants meet this criterion before training ended.

The untrained symmetry relations were the equivalence relations: B-C and C-B relations and the symmetry relations: Sample comparisons B-A and C-A relations. These two conditions were assessed over 36 trials (18 of equivalence and 18 of symmetry) without feedback. Participants were classified as having “passed” derived relations training if they responded correctly to at least 32 of the 36 trials in this condition. All 12 participants in the Near Miss = Loss condition, and all 12 participants in the Near Miss = Almost condition “passed” both equivalence and symmetry. There was no discernible difference between either condition in the speed and accuracy in which participants passed.

### Experimental Manipulation

The experimental manipulation between groups involved the pairing of verbal labels with gambling outcomes during the derived relations training phase. This design tested whether changing the verbal association of near-misses would alter subsequent attentional bias toward these outcomes. Specifically:

#### Near-Miss = Loss Condition

**Fig. 3:**
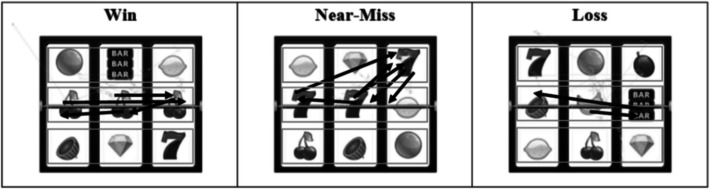
Visual displays of the three types of events with interest areas. Arrows show an example of the direction of saccadic eye-movements

Participants in this condition learned to associate the word "LOSS" with near-miss outcomes (i.e., two matching symbols with one offset: see bananas in the slot machine outcome of the bottom right panel of Fig. 3). This pairing emphasized the objective reality that near-misses are losing outcomes, despite their perceptual similarity to wins. As shown in Fig. 1, the complete training protocol for the near-miss = loss condition involved:

- Pairing abstract symbol A1 with the word "WIN" and images of winning slot outcomes
- Pairing abstract symbol A2 with the word "LOSS" and images of near-miss slot outcomes.
- Pairing abstract symbol A3 with the word "ALMOST" and images of complete loss outcomes

#### Near-Miss = Almost Condition

Participants in this condition learned to associate the word "ALMOST" with near-miss outcomes, reinforcing the common gambler perception that these outcomes represent "almost winning" (this time the near-miss bananas result is at the bottom of the middle column in Fig. 2). As shown in Fig. 2, the complete training protocol involved:

- Pairing abstract symbol A1 with the word "WIN" and images of winning slot outcomes
- Pairing abstract symbol A2 with the word "ALMOST" and images of near-miss slot outcomes.
- Pairing abstract symbol A3 with the word "LOSS" and images of complete loss outcomes

This manipulation tested whether the verbal framing of near-misses influences their attention-capturing properties, with implications for understanding how cognitive interpretations shape gambling behavior.

### Research Design

A 2 × 2 mixed between-within research design was used. Participants were randomly assigned to either the “near-miss = almost” group or the “near-miss = loss” group. All participants completed the tasks twice, and in between, they completed the verbal training task. Two dependent variables were employed. The first was direction bias to near-misses. That is, the proportion of eye-movements towards the near-miss event (the identical off-centre symbol) compared to eye-movements away from the centre line during any other event. The second was duration bias to near-misses. That is, the proportion of total eye-gaze time on the near-miss outcome compared to total eye-gaze time on the centre line only.

### Measures of Attentional Bias

Based on pilot testing observations that revealed participants' eye movements predominantly followed the centre payline when checking for wins, the measurement approach for the two dependent variables was established. The two attentional bias dependent variables were operationalized as follows:

1. **Direction bias (attentional capture):** The frequency of saccades directed toward the near-miss symbol location (i.e., the offset symbol on the top or bottom row) when a near-miss outcome was displayed, expressed as a proportion of total near-miss trials (range: 0–36).
2. **Duration bias (attentional maintenance):** The proportion of total fixation time spent on the near-miss symbol location relative to total fixation time across all interest areas during near-miss trials.

These measures remained consistent across both testing phases (pre- and post-training) and were not altered during the study. The pilot testing simply informed how these attentional processes would be most effectively measured.

Three areas of interest were set up to observe these eye movements. Each area measured approximately the height of the individual symbols in the display and the length of each line. Trials during which a saccade was greater than 3 degrees and reached the top or bottom interest area were deemed valid and were counted towards dependent measures.

## Results

Prior to examining the effects of verbal training on attentional bias, preliminary analyses were conducted to ensure data integrity and examine baseline characteristics. No univariate outliers were detected across any dependent variable (all Z-scores < ± 3.29), and Shapiro–Wilk tests confirmed that assumptions of normality were satisfied for all variable distributions (*p* > 0.05). Levene's tests indicated homogeneity of variance across groups for both outcome measures (*p* > 0.05). Table 1 presents descriptive statistics for both attentional bias measures (direction bias and duration bias) as a function of experimental condition and time of assessment.

**Table 1:** Means and Standard Deviations for Attentional Bias Measures by Condition and Time

Measure	Condition	Pre-training	Post-training	Change Score
**Direction Bias**	Near-miss = Almost	M = 10.17 (SD = 4.61)	M = 14.17 (SD = 5.84)	M = −4.00 (SE = 1.46
	Near-miss = Loss	M = 11.25 (SD = 5.03)	M = 6.00 (SD = 3.64)	M = + 5.25 (SE = 1.46)
**Duration Bias (ms)**	Near-miss = Almost	M = 489.25 (SD = 313.61)	M = 760.25 (SD = 354.22)	M = −271 (SE = 68.34)
	Near-miss = Loss	M = 646.17 (SD = 307.07)	M = 450.75 (SD = 251.85)	M = 195.42 (SE = 68.34)

Direction bias scores represent the number of eye-movement saccades toward near-miss symbols out of 36 possible trials. Duration bias represents mean gaze fixation time in milliseconds on near-miss interest areas

As shown in the table, the two groups demonstrated comparable baseline levels of attentional bias during the pre-training phase. However, marked divergence emerged following the verbal training manipulation, with the pattern of change differing systematically between conditions. These descriptive patterns were then subjected to formal statistical analysis of variance (ANOVA) to test the primary hypotheses regarding the malleability of near-miss attentional bias through derived relational training.

### Direction Bias

A 2 × 2 mixed between-within ANOVA was performed to evaluate the differences between the number of eye-movements towards near-win stimuli according to the type of training (1 = almost training; 2 = loss training) compared to before and after the training. No univariate outliers were detected (Z ± 3.29, *p* < 0.001) and Shapiro–Wilk’s test confirmed the assumption of normality was met for all independent-dependent combinations (*p* > 0.05). Levene’s test indicated that the assumption of homogeneity of variances was not-violated (*p* > 0.05).

**Fig. 4:**
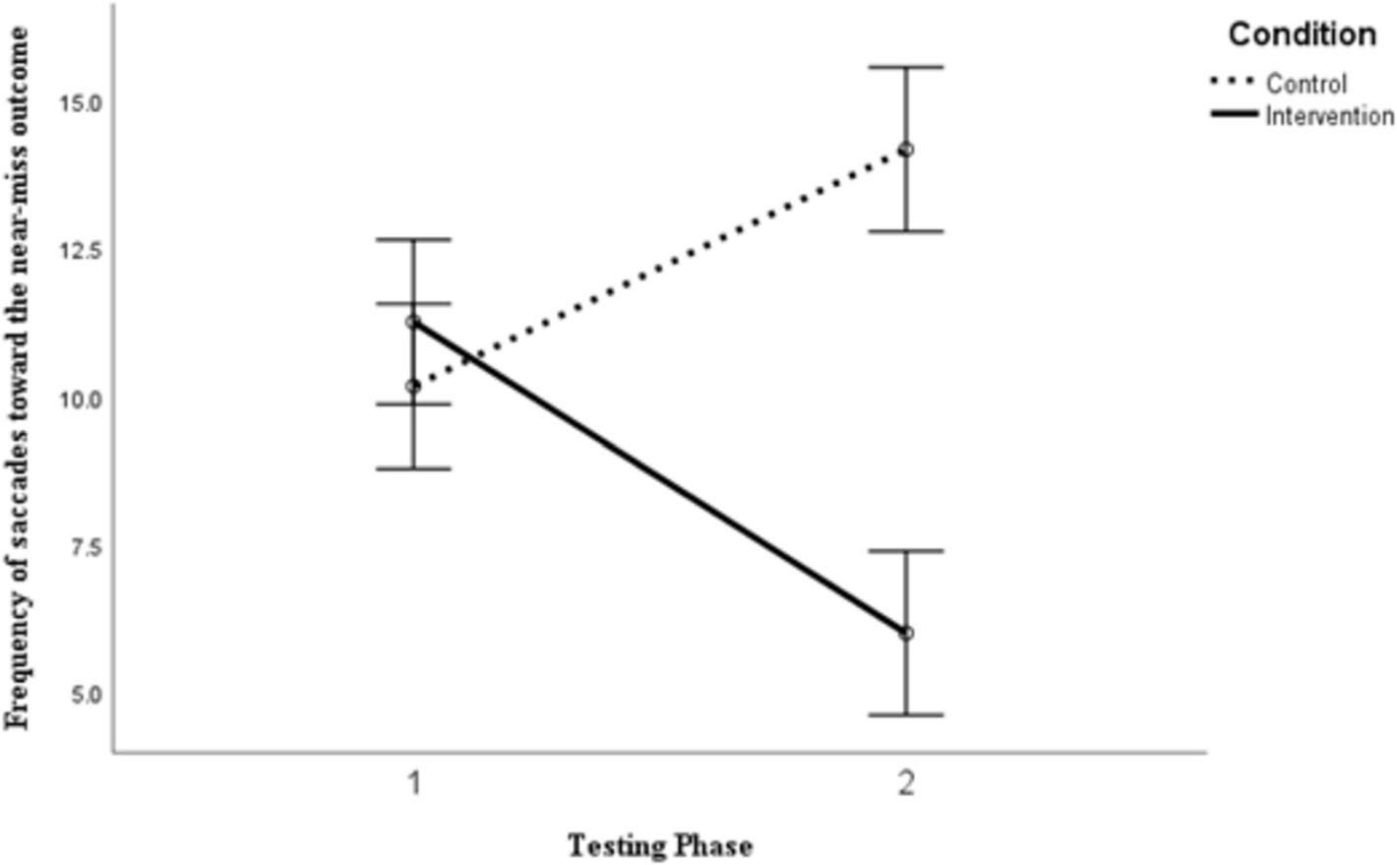
The effects of verbal training and time on direction bias

The ANOVA revealed a significant interaction effect between training and time of testing *F* (1, 22) = 20.16, *p* < 0.001, η^2^_p_ = 0.478. The between-subjects main effect of condition showed that the difference between the groups was statistically significant, *F* (1, 22) = 4.48, *p* = 0.046, η^2^_p_ = 0.169. However, the repeated measures main effect of time was not statistically significantly different, *F* (1, 22) = 0.37, *p* = 0.550, η^2^_p_ = 0.016. Visual examination of the unambiguous differences (see Fig. 4) shows that after the intervention those who were trained to learn that near-misses are a loss made significantly fewer eye-movements towards near-miss stimuli than those who were trained that a near-miss is an almost event.

### Duration Bias

A 2 × 2 mixed between-within ANOVA was performed to evaluate whether there was a difference between the duration of eye-movements focused on near-miss stimuli depending on type of training (1 = almost training; 2 = loss training) and whether this changed between before and after the training. No univariate outliers were detected (Z ± 3.29, *p* < 0.001) and Shapiro–Wilk’s test confirmed the assumption of normality was met for all independent-dependent combinations (*p* > 0.05). Levene’s test indicated that the assumption of homogeneity of variances was not-violated (*p* > 0.05).

**Fig. 5:**
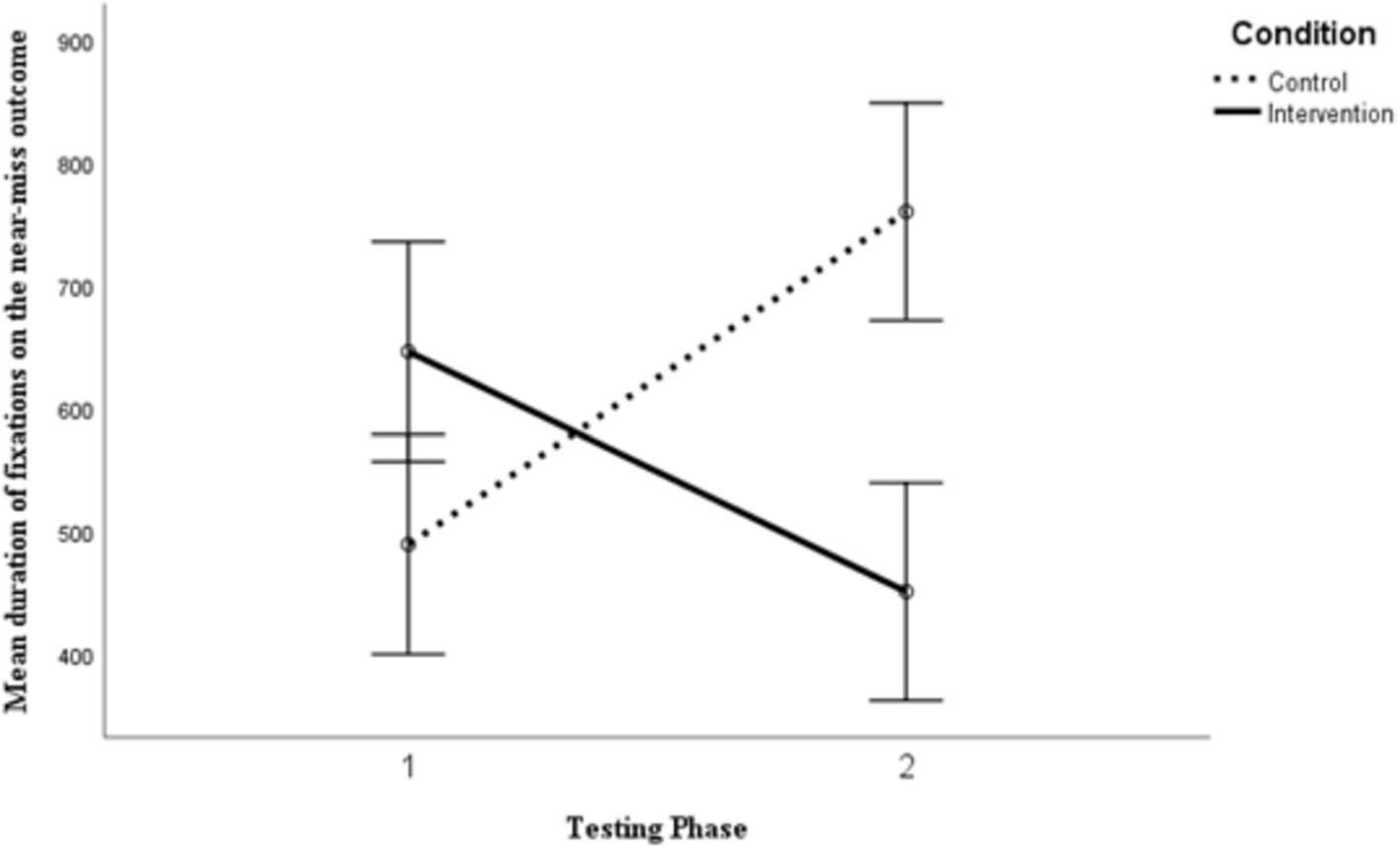
The effects of verbal training and time on duration bias

The ANOVA revealed a significant interaction effect between training and time of testing *F* (1, 22) = 23.29, *p* < 0.001, η^2^_p_ = 0.514. The between-subjects main effect of condition showed that the difference between the groups was statistically significant, *F* (1, 22) = 101.5, *p* < 0.001, η^2^_p_ = 0.822. However, the repeated measures main effect of time was not statistically significantly different, *F* (1, 22) = 0.61, *p* = 0.442 η^2^_p_ = 0.027. Visual examination of the unambiguous differences (see Fig. 5) shows that after the intervention those who were trained to learn near-misses are a loss, looked at the near-miss stimuli for less time than those who were trained that a near-miss is an almost event.

## Discussion

This study aimed to investigate whether gamblers’ responses to near-misses could be modified by instructing them that near-misses are equivalent to losses. It was hypothesized that participants who internalized the "near-miss = loss" relation would demonstrate a reduced attentional bias towards near-miss outcomes. The theoretical framework guiding this study rested on two key assumptions. First, the near-miss effect in gambling is understood as a learned behavior derived from everyday life, which is subsequently misapplied in gambling contexts (Dymond et al., 2012). Second, sensitization of the dopaminergic reward system can lead individuals to develop an increased attentional bias towards relevant stimuli (Ciccarelli et al., 2016; Robinson & Berridge, 2008). Specifically, near-misses are believed to be stimulus events that capture and maintain the attention of sensitized gamblers (Grant & Bowling, 2015). Therefore, it was anticipated that recreational gamblers would exhibit reduced attentional engagement and maintenance towards near-misses following the training of the "near-miss = loss" relation.

The results of this study support the hypothesis: participants who learned to associate near-misses with the concept of a loss showed a significant reduction in attentional bias towards near-misses compared to those who associated near-misses with the concept of "almost." This reduction in attentional bias was observed both in terms of attentional engagement and maintenance. These findings have important clinical implications, suggesting a potential method for reducing the incentive-sensitization for near-misses, which could lead to a decrease in gambling behavior.

Two primary theories compete in explaining the near-miss effect. The cognitive perspective posits that a flaw in the gambler's personality leads to faulty cognition, where a near-miss is perceived as an indicator that a win is imminent (Ciccarelli et al., 2016). Conversely, the behavioral perspective argues that responses to near-misses are maintained verbally, with disordered gamblers erroneously learning that near-miss outcomes are nearly wins, thereby reinforcing their behavior (Dixon et al., 2009; Tan et al., 2015). While the current findings strongly align with the latter perspective, it is proposed that the near-miss effect is governed by an interplay between cognitive and verbal processes, best understood within the incentive-sensitization model. Specifically, the psychological desire (or "wanting") elicited by a cue is as crucial as the behavioral response itself. Modifying the meaning of the cue, therefore, should reduce the preferential attention towards near-misses, leading to a decrease in gambling behavior.

This study's findings further demonstrate that the near-miss effect is a stimulus capable of capturing and maintaining attention, consistent with prior research on cognitive biases and preferential attention in gambling. For example, Grant and Bowling (2015) found that an increase in faulty gambling beliefs was associated with increased attentional bias towards gambling-related stimuli. Similarly, Ciccarelli et al. (2016) reported that problem gamblers exhibited heightened attentional engagement compared to non-problem gamblers, alongside greater levels of craving. The present study adds to this body of work by showing that near-misses capture and hold the attention of all participants initially, thus reinforcing the idea that changes in attentional bias towards gambling stimuli are a significant factor in the development of disordered gambling. Moreover, the increased craving reported by disordered gamblers in Ciccarelli et al.'s study suggests that attentional bias towards near-misses may be indicative of heightened craving. Encouragingly, the results of this study suggest that this subjective craving, triggered by gambling, can be mitigated through appropriate training.

The primary objective of this study was to determine whether an attentional bias towards near-misses is verbally maintained, and the results support this hypothesis. At the second time point, the group instructed that "near-miss = loss" exhibited reduced attentional engagement and maintenance towards near-miss outcomes compared to their initial responses and in comparison, to the group trained to associate near-misses with "almost." These findings add to the growing body of evidence (Dixon et al., 2009; Tan et al., 2015) suggesting that verbal processes are instrumental in controlling the near-miss effect and that cognitive biases in gambling are not rooted in faulty personality traits but are instead learned responses that can be modified through changes in verbal associations. Notably, earlier research on near-misses often relied on self-report measures to assess the perceived significance of near-misses. In contrast, this study employed an implicit and more objective measure, enhancing confidence in the validity and reliability of the findings.

Given that near-misses are a cognitive distortion and do not objectively predict wins (Griffiths, 1999), each bet on an EGM should be treated independently by the player: the probability of winning remains constant regardless of previous outcomes, including near-misses (Turner, 2011; Harrigan & Dixon, 2010). This mathematical independence means near-misses provide no predictive information about future spins, making the persistent belief in their predictive value a fundamental misunderstanding of EGM mechanics. Despite the mathematical reality that near-misses do not increase the likelihood of future wins, their motivational pull persists, indicating that cognitive understanding alone may be insufficient to counteract their reinforcing effects. This dissociation between intellectual knowledge and emotional response highlights the potential value of interventions targeting automatic attentional processes—such as the verbal relabelling demonstrated in this study.

The clinical implications of these findings are threefold. First, the demonstration that verbal relabelling can modify attentional bias suggests a potential brief intervention strategy for gambling treatment. Clinicians could implement psychoeducational modules teaching clients to mentally relabel near-misses as "losses" rather than "almost wins," potentially reducing the reinforcing properties of these events (Dixon et al., 2009). Second, our eye-tracking methodology could serve as an objective assessment tool for measuring treatment progress, as reductions in attentional bias may indicate decreased vulnerability to near-miss effects (Ciccarelli et al., 2016). Third, these findings support the integration of cognitive restructuring techniques targeting near-miss interpretations within existing cognitive-behavioral therapies for gambling disorder. For example, therapists could use visual demonstrations of slot machine mechanics while explicitly training clients to recognize and relabel near-misses, similar to the derived relations training employed in this study. Such interventions could be particularly valuable during early treatment phases when clients are learning to identify and challenge gambling-related cognitive distortions (Fortune & Goodie, 2012).

The generalizability of these findings is somewhat constrained by the sample used in this study, which consisted exclusively of recreational gamblers. While this sample is consistent with previous research (Dixon et al., 2009; Tan et al., 2015), future research should examine whether these effects can be replicated in more frequent gamblers. Studying heavier gamblers would be particularly valuable, as the current study showed that all participants successfully acquired the trained relations. However, individuals with greater gambling exposure might struggle to acquire these relations, suggesting that not all gamblers may be so efficiently instructed. Future studies should replicate this research using a sample of problem gambling participants, as measured by the Problem Gambling Severity Index (Smith & Wynne, 2002) and related sample of heavy consumption gamblers as measured by the Consumption Screen for Problem Gambling (Rockloff, 2012), who primarily play EGMs. As EGM results are entirely mathematically predetermined—luck based—near-misses have no bearing on future results. If problem/heavy gamblers can successfully learn the "near-miss = loss" relation, the findings of this study should replicate, offering valuable insights support for incentive-sensitization theory and in applied settings as an efficient intervention targeting gambling disorders.
